# Supplementary material for: Hydroxychloroquine Mitigates Dilated Cardiomyopathy Phenotype in Transgenic D94A Mice
Source: Int J Mol Sci. 2022 Dec 9;23(24):15589. doi: 10.3390/ijms232415589 (PMC9779604; doi:10.3390/ijms232415589)
Supplement: Supplementary file 1 [file ijms-23-15589-s001.zip › ijms-2033187-supplementary.pdf]

International Journal of
Molecular Sciences

MDPI

Article

Hydroxychloroquine Mitigates Dilated Cardiomyopathy Phenotype in Transgenic D94A Mice

Rosemeire M. Kanashiro-Takeuchi [1,2], Katarzyna Kazmierczak [1], Jingsheng Liang [1], Lauro M. Takeuchi [2], Yoel H. Sitbon [1] and Danuta Szczesna-Cordary [1,*]

Supplementary Materials:

Supplemental tables

Table S1. Doppler echocardiographic evaluation of Tg-D94A and NTg mice at baseline and 30 days after treatment with HCQ or placebo

	BASELINE		DAY 30			
			HCQ		Placebo	
**Parameter**	**Tg-D94A**	**NTg**	**Tg-D94A**	**NTg**	**Tg-D94A**	**NTg**
N° of animals	13	13	8	8	6	10
(Males, Females)	(6, 7)	(6, 7)	(4, 4)	(4, 4)	(3, 3)	(5, 5)
Mitral Pulsed Wave Doppler:						
IVRT (ms)	13.6 ± 0.5	14.5 ± 0.4	13.3 ± 1.0	15.3 ± 0.5	13.0 ± 0.8	13.8 ± 0.7
MV E/A	1.6 ± 0.1	1.5 ± 0.07	1.5 ± 0.1	1.4 ± 0.1	1.5 ± 0.3	1.4 ± 0.1
LV MPI	0.6 ± 0.03	0.6 ± 0.03	0.7 ± 0.04	0.7 ± 0.04	0.6 ± 0.05	0.6 ± 0.03
MV E/E'	-26 ± 1.4	-28.7 ± 2.0	-32.2 ± 2.3	-30.5 ± 1.9	-29.3 ± 2.5	-25.0 ± 1.6
Pulmonary Pulsed Wave Doppler:						
PAT/PET	0.3 ± 0.02	0.4 ± 0.05	0.3 ± 0.0	0.3 ± 0.02	0.3 ± 0.0	0.4 ± 0.02

Data are presented as mean ± SEM of n = Nº of animals with significance calculated by unpaired t-test to compare Tg-D94A vs. NTg at baseline and mixed-effects analysis followed by Sidak's multiple comparisons test for groups treated with HCQ vs. placebo. No significant differences between the groups and/or treatments were observed. Abbreviations: IVRT, isovolumetric relaxation time; E/A ratio, the ratio of early to late mitral inflow velocity; LV MPI, LV myocardial performance index; E/E′ ratio, the ratio between early mitral inflow velocity and mitral annular early diastolic tissue velocity; and PAT/PET, the ratio between pulmonary acceleration time and pulmonary ejection time.

Table S2. Hemodynamic parameters and indices of systolic and diastolic function derived from left ventricle pressure-volume (PV) loops in HCQ-treated and placebo-treated Tg-D94A vs. NTg mice

	HCQ		Placebo	
Parameter	Tg-D94A	NTg	Tg-D94A	NTg
N° of animals<br>(Males, Females)	4<br>(2, 2)	6<br>(4, 2)	6<br>(3, 3)	5<br>(2, 3)
HR (bpm)	534 ± 10.2	540 ± 8.5	534 ± 8.2	525 ± 4.3
Integrated Performance:				
EF (%)	60.2 ± 7.5	60.4 ± 5.0	59.7 ± 8.5	49.8 ± 4.3
SW (mmHg x ml)	2498 ± 781	2078 ± 133	2583 ± 579	2232 ± 380
SV (ml)	35.9± 9.3	29.2 ± 1.6	30.9 ± 6.2	29.2 ±. 1.6
CO (ml/min)	19.2 ± 5.1	15.2± 0.7	16.5 ± 3.3	15.1 ± 1.8
Ea/Ees	2.3 ± 0.3	2.5 ± 0.5	4.0 ± 0.8*	1.8 ± 0.4
Afterload:				
LVESP (mmHg)	95.6 ± 9.3	95.5 ± 6.4	114 ± 5.5	97.2 ± 8.5
Ea (mmHg/ml)	3.1 ± 0.6	3.3 ± 0.4	4.5 ± 0.9	3.5 ± 0.5
Preload:				
LVEDP (mmHg)	20.5 ± 4.4	15.9 ± 3.6	20.8 ± 5.3	22.2 ± 10.7
LVEDV (ml)	62.2 ± 14.0	50.4 ± 4.4	50.0 ± 6.3	53.1 ± 2.1
Contractility:				
dP/dT_{max} (mmHg/s)	6795 ± 753	6818 ± 663	7940 ± 777	6891 ± 468
dP/dT_{max}_EDV (mmHg/s per ml)	80.7 ± 15.5	109.9 ± 40.9	99.4 ± 26.4	97.4 ± 14.2
Ees (mmHg/ml)	1.4 ± 0.3	1.5 ± 0.2	1.4 ± 0.3	2.6 ± 0.7
PRSW (mmHg)	46 ± 7.4	45 ± 6.5	51 ± 8.1	45 ± 10.1
P@dP/dT max(mmHg)	57.9 ± 6.7†	56.7 ± 5.8	72.7 ± 2.7**	62.5 ± 0.9
Lusitropy:				
dP/dT_{min} (mmHg/s)	-5648 ± 965	-5769 ± 768	-6920 ± 965	-6665 ± 881
EDPVR (exponential)	0.018± 0.005	0.017 ± 0.006	0.014 ± 0.003	0. 010 ± 0.0
Tau (ms)	11 ±1.0	10.4 ± 1.2	10.8 ± 1.7	10.6 ± 1.3

Data are presented as mean ± SEM of n = Nº of animals with significance calculated by unpaired t-test with significance *p<0.05 and **p<0.01 for Tg-D94A placebo vs. NTg placebo, and †p<0.05 for Tg-D94A HCQ vs. Tg-D94A placebo). Abbreviations: HR, heart rate; EF, ejection fraction; SW, stroke work; SV, stroke volume; CO, cardiac output; Ea, arterial elastance; Ea/Ees, the ratio between Ea and slope of end-systolic pressure-volume relationship (slope ESPVR or Ees); LVSP, LV systolic pressure; LVED, LV end-diastolic volume; dP/dT max, peak rate for pressure rise; dP/dTmax_EDV, peak rate for pressure rise by end-diastolic volume; PRSW, preload recruitable stroke work; P@dP/dTmax, pressure at maximum dP/dT; dP/dTmin, peak rate for pressure decline; EDPVR, slope of end-diastolic pressure-volume relationship, and Tau, relaxation time constant.

Supplemental figures

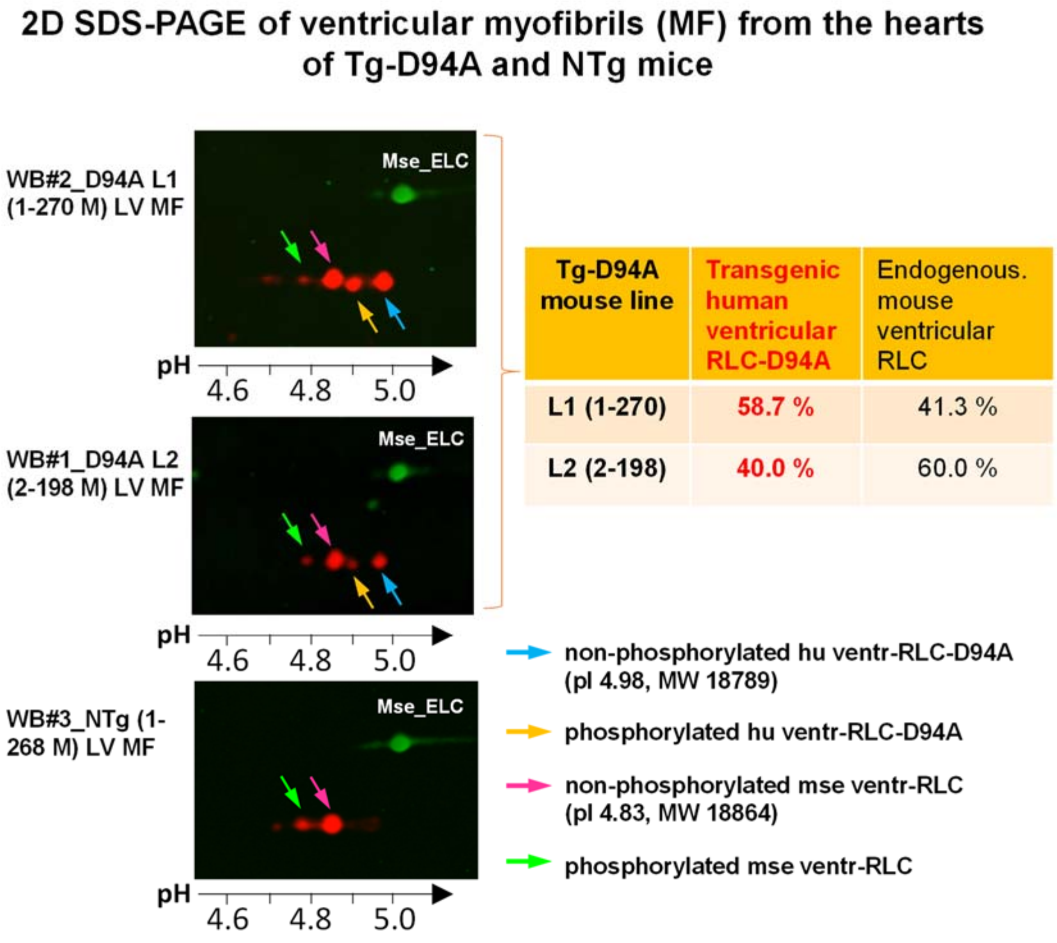

Figure S1. Expression of human ventricular D94A-RLC protein in left ventricles (LV) of Tg-D94A mice. Percent of D94A mutant expression was assessed using two-dimensional (2D) SDS-PAGE in myofibrils (MF) isolated from LV of Tg-D94A mice (L1 and L2). Myofibrils from NTg mice were used as controls. A clear separation of the human vs. endogenous mouse (mse) ventricular isoforms of the RLC was assessed via isoelectric focusing. MF were prepared from LV of mouse hearts and processed using 2D clean-up kit (Cytiva, Marlborough, MA, USA) to remove interfering substances such as salts, lipids, nucleic acids, and detergents), and then resuspended in IEF buffer containing 8 M urea (USB Corporation, Cleveland, OH, USA), 2% CHAPS (Sigma-Aldrich, St. Louis, MO, USA), 0.5% carrier ampholyte (GE Healthcare, Uppsala, Sweden), 40 mM DTT (Bioworld molecular life sciences, Dublin, OH, USA), and 0.002% bromophenol blue (Bio-Rad Laboratories, Hercules, CA, USA). Samples were subjected to first dimension-isoelectric focusing using Immobiline DryStrip gels (IPG strips, pH 4–7) (GE Healthcare, Uppsala, Sweden) followed by the 2nd dimension–SDS-PAGE. RLC isoforms were detected by Western blots labeled with polyclonal RLC CT-1 antibodies (produced in this lab [1]), followed by a goat anti-rabbit antibody conjugated with the fluorescent dye IR red 800 (red dots) (Rockland, Pottstown, PA, USA). The mouse isoform of myosin essential light chain (Mse_ELC) was used as a loading control. It was detected with an ab680 antibody (Abcam, Boston, MA, USA) followed by a goat anti-mouse secondary antibody labeled with the fluorescent dye Cy5 (shown in green) (Rockland, Pottstown, PA, USA). Bands intensities were quantified using ImageJ (imagej.nih.gov/ij).

1. Wang, Y.; Xu, Y.; Kerrick, W. G. L.; Wang, Y.; Guzman, G.; Diaz-Perez, Z.; Szczesna-Cordary, D., Prolonged Ca^{2+} and force transients in myosin RLC transgenic mouse fibers expressing malignant and benign FHC mutations. *J Mol Biol* **2006,** 361, (2), 286-299.
